# Metabolic Status and Expression Level of SREBP mRNA and Mir‐33 Among Children Conceived by Assisted Reproductive Technology

**DOI:** 10.1155/ijog/2271298

**Published:** 2025-11-29

**Authors:** Yifan Cui, Yuqian Wang, Xiaoxia Wang, Yaping Liu, Panpan Zhang, Dan Hu, Xuwu Xiao

**Affiliations:** ^1^ Department of Graduate, Dalian Medical University, Dalian, Liaoning, China, dlmedu.edu.cn; ^2^ Department of Child Health Care, Dalian Women and Children′s Medical Group, Dalian, Liaoning, China; ^3^ Department of Pediatrics, The Second Affiliated Hospital of Dalian Medical University, Dalian, Liaoning, China, dlmedu.edu.cn

**Keywords:** assisted reproductive technology, metabolic status, miR-33, preschool-aged children, SREBP mRNA

## Abstract

**Background:**

Due to the growth in the global consumption of assisted reproductive technology (ART), it is possible that long‐term health impacts on offspring have come into focus. ART has offered a welcome solution to infertility, but the fear has been on its effect on the metabolic health of children born on their behalf. Past studies indicate that ART‐conceived individuals can have characteristic metabolic profiles relative to their naturally conceived (NC) peers and are therefore potentially predisposed to changes in lipid and glucose handling. Physiopathological glycolipid metabolism, a hallmark of cardiometabolic health, is believed to be modulated not only by environmental and other external factors but also by intracellular regulation proteins, including sterol regulatory element‐binding protein (SREBP) and miR‐33, although there is little evidence on the effects of ART on these regulatory pathways in early childhood.

**Objective:**

This paper sought to compare the glycolipid metabolic profile of the kids who are in preschool age and who were conceived through ART and kids who were NC. The second aim was to study the expression of SREBP‐1/2 and miR‐33 in peripheral blood and the possible nature of the role of these players in regulating early‐life metabolism.

**Subject and Methodology:**

A total of 220 children aged between 3 and 6 years were recruited of which complete data has been obtained from 206 children out of 98 that were conceived via in vitro fertilization/intracytoplasmic sperm injection (ICSI) (ART group) and 108 that were conceived naturally (NC group). Anthropometric measures—such as body weight, height, and waist circumference—to determine physical growth and obesity status were taken. Biochemical variables, triglyceride (TG), high‐density lipoprotein cholesterol (HDL‐C), apolipoprotein A1 (ApoA1), apolipoprotein B (ApoB), fasting serum insulin (FINS), and homeostatic model assessment of insulin resistance (HOMA‐IR) were determined. A centrifugal column was used to obtain peripheral blood RNA, and relative gene expression levels of SREBP‐1, SREBP‐2, miR‐33a, and miR‐33b were measured by qPCR.

**Results:**

Compared with the IVF group, children in the ICSI group had significantly lower weight, height, and waist circumference (*p* < 0.05). In contrast, children in the NC group had significantly lower weight, BMI, and waist circumference, with all differences being statistically significant (*p* < 0.05). Additionally, compared with the IVF group, the NC group showed significantly increased levels of HDL‐C and ApoA1 (*p* < 0.05). Meanwhile, the ICSI group had significantly higher ApoB levels and HOMA‐IR scores (*p* < 0.05). Compared with the NC group, children in the ICSI group showed significantly higher levels of TG, ApoB, FINS, and HOMA‐IR (*p* < 0.05), while ApoA1 levels were significantly lower (*p* < 0.05). When comparing clinical outcomes related to blood pressure, blood glucose, and blood lipids for abnormalities, there was no difference in incidence between the ART group and the NC group (*p* > 0.05). In addition, the expression of peripheral blood SREBP‐1 mRNA, SREBP‐2 mRNA, miR‐33a, and miR‐33b was compared. Compared with the IVF group, SREBP‐1 mRNA and miR‐33b levels in the NC group were significantly reduced, while miR‐33a levels in the ICSI group were significantly decreased (*p* < 0.05). Compared with the NC group, SREBP‐1 mRNA and SREBP‐2 mRNA were significantly increased in the ICSI group (*p* < 0.05), while miR‐33a was significantly decreased (*p* < 0.05). Correlation analysis showed that in the ART group, SREBP‐1 mRNA expression levels were positively correlated with BMI, ApoB, fasting blood glucose, FINS, and HOMA‐IR and negatively correlated with ApoA1. SREBP‐2 mRNA levels were positively correlated with HOMA‐IR and negatively correlated with ApoA1. Additionally, the expression levels of miR‐33a were negatively correlated with HDL‐C, while miR‐33b levels were negatively correlated with both HDL‐C and FINS.

**Conclusion:**

Our data suggest that although children born by means of ART are otherwise normal in their glycolipid metabolism, they are more prone to overweight and obesity and have different biochemical and molecular characteristics than NC children. The upregulation of miR‐33b, SREBP‐1, and SREBP‐2 observed indicates that ART can play a role in regulating the process of glycolipid metabolism during early childhood at a molecular level. Such alterations might not present the form of a blatant metabolic condition at this age but may consist of initial symptoms of future troublesome metabolic health. Prolonged follow‐up of the ART offspring and additional mechanistic work are desirable to be able to determine whether these early changes are the underlying reasons behind higher metabolic risk as adults.

## 1. Introduction

Infertility is one of the three major diseases impacting human life and health in the 21st century. The emergence and advancement of assisted reproductive technology (ART) have provided hope for infertile couples to conceive children [1]. ART is one of the most effective ways to treat infertility. ART includes fertility treatment in which either eggs or embryos are handled outside a female’s body to promote successful pregnancies and healthy offspring [2]. ART involves a set of in vitro procedures executed in a specific order, collectively called an ART cycle: stimulating the ovaries, surgically retrieving eggs, fertilizing them with sperm in a lab, and returning the embryo(s) [3, 4]. Current ART procedures encompass in vitro fertilization (IVF) with or without intracytoplasmic sperm injection. In recent years, over 390,000 children conceived through ART are born annually worldwide [5], and since the introduction of ART, over 10 million children brought into existence via this technology have come into existence [6]. According to the Developmental Origins of Health and Disease (DOHaD) hypothesis, environmental exposures during early life stages may predispose offspring to an increased risk of developing various diseases later in life [7]. Unlike natural conception, assisted reproduction bypasses the natural sperm selection process that occurs during normal reproduction and may involve delayed fertilization. Furthermore, embryos undergo exposure to nonphysiological environments, including in vitro manipulations and culture media, which could potentially affect the growth, metabolism, and other developmental aspects of the offspring.

Recent population‐based studies indicate that children conceived via ART show notably elevated fasting blood glucose levels, serum insulin levels, indices of insulin resistance, and carotid intima‐media thickness [8]. In animal research, Fang et al. investigated ART mice and discovered that female ART mice exhibited significantly reduced amounts of high‐density lipoprotein cholesterol (HDL‐C) and Apo‐A1, along with a noteworthy rise in LDL‐C levels [9]. Metabolic disorders can lead to obesity and diabetes, as well as cardiovascular and cerebrovascular diseases, posing a significant global public health challenge. Metabolic issues during childhood may persist in adulthood; however, metabolic syndrome in children is often reversible and can be prevented through early diagnosis, appropriate monitoring, and effective intervention [10]. The current investigation seeks to explore the distinctions in metabolic profiles between kids developed via ART and those developed via NC. Additionally, if variations exist, we aim to identify the specific aspects in which these disparities manifest. Metabolism‐related indicators were mainly monitored in this study including triglyceride (TG), HDL‐C, serum apolipoprotein A1 (ApoA1), serum apolipoprotein B (ApoB), fasting serum insulin (FINS), and homeostatic model assessment of insulin resistance (HOMA‐IR).

Recent investigations have shown that SREBP is essential in human glycolipid metabolism [11–14]. The SREBPs are key transcription regulators of genes involved in cholesterol biosynthesis and uptake. MicroRNAs (miR‐33a/b) embedded within introns of the SREBP genes target the adenosine triphosphate‐binding cassette transporter A1 (ABCA1), an important regulator of high‐density lipoprotein (HDL) synthesis and reverse cholesterol transport [15]. As a newly identified hepatogenic secretory protein, SREBP is primarily distributed in the liver and serum, advancing the expansion of pancreatic *β* cells through the growth/survival pathway [16]. The SREBP family of transcription elements controls the expression of genes that undertake a crucial part in the development of fatty acids, cholesterol, and various lipid components [17]. This study investigates the roles of peripheral blood SREBP mRNA and the miR‐33 gene, which is formed through intron cleavage, in the glycolipid metabolism of children conceived through ART. Our findings seek to establish a rigorous scientific basis for comprehending the potential mechanisms that contribute to metabolic concerns for kids produced by ART, alongside the identification and precise management of metabolic disorders.

## 2. Method

### 2.1. Study Population

This research engaged 110 children aged 3–6 years, all conceived via IVF/ICSI, at the Dalian Women and Children’s Medical Group during the period from January 2024 to January 2025. The group for comparison comprised 110 children conceived naturally, all within the same age range, who attended the same clinic during the identical timeframe. The ultimate evaluation encompassed an aggregate of 206 children for whom full information (including pregnancy status, gestational age, and birth status of the two groups of children was collected and documented by trained professionals) was available. Among the excluded participants, 14 children were removed due to nonfasting venous blood samples. The final cohort included 98 children conceived via IVF/ICSI and 108 children conceived through natural means. Children in both groups were born between January 2018 and January 2021. Children with severe congenital diseases, inherited metabolic disorders, liver conditions, or a gestational age of ≤ 32 weeks were excluded from the study. In addition, children and their mothers included in the study who have a history of diabetes and a controlled diet were also excluded. For children conceived naturally, the socioeconomic background of ART children (e.g., same or similar kindergarten and residential address) should be taken into account as much as possible. Informed consent, duly documented in writing, was obtained from all children and their parents, receiving the endorsement of the Ethics Committee of Dalian Women and Children’s Medical Group (2023007).

### 2.2. Data Collection

All physical measurements and assessments were conducted by professionally trained nurses at the Dalian Women and Children’s Group. A calibrated electronic height and weight scale was used to measure height (to within 0.1 cm) and weight (to within 0.1 kg). A measuring tape was employed to ascertain waist circumference. Each measurement was taken two times, with the average result documented. Blood pressure on the right arm was measured three times while the child was in a seated position, using a clinically calibrated electronic sphygmomanometer (Model YE8900A, China), and the average of the last two values was utilized for evaluation. Measurement data was carried out by professionals, who recorded specific information for each included publication in predesigned tables.

Following a minimum of 8 h of a fast, specimens of blood were collected from each participant from 7:30 to 9:30 a.m. Two blood samples were obtained in total. A sample of approximately 3 mL was put in a vacuum blood collection tube composed of separation gel/coagulant PET material (yellow), while another sample of approximately 3 mL was placed in an EDTA‐K2 glass vacuum blood collection tube (purple). The yellow blood collection tube samples were analyzed using an automatic biochemical analyzer (Labospect 008 AS, Japan) to determine FBG, TG, TC, LDL‐C, HDL‐C, ApoA1, and ApoB. FINS levels were quantified utilizing a chemiluminescence immunoassay analyzer (Roche E601, Germany). The evaluation of insulin sensitivity was conducted through the use of HOMA‐IR. The calculation of HOMA‐IR was performed employing HOMA − IR = fasting glucose (mmol/L)∗fasting insulin (uU/mL)/22.5.

Purple blood collection tube samples were used for the extraction of SREBP mRNA and miR‐33. All gene extractions were performed within 2 h of blood sample collection on the same day. The Steady Pure Blood, Serum, and Plasma Small RNA Extraction Kit (AG21030) was utilized to extract total RNA using the centrifugal column method. The extraction process adhered strictly to the manufacturer’s guidelines for total RNA purification, ensuring that all specified steps were meticulously followed. The M‐MLV Reverse Transcriptase Reverse Transcription Kit was utilized for RNA reverse transcription, adhering to the manufacturer’s guidelines. This process involved a two‐step protocol that included the removal of gDNA followed by reverse transcription. The Steady Pure Blood, Serum, and Plasma Small RNA Extraction Kit (AG21030) was employed to isolate total RNA, including small RNA, using the centrifugal column method. The extraction process strictly adhered to the small RNA purification steps outlined in the manufacturer’s procedure. The reverse transcription of miRNA was done with the miRNA 1st Strand cDNA Synthesis Kit (AG11717), complying with the specifications set forth by the manufacturer for A‐tailed miRNA 1st strand cDNA synthesis.

Quantitative PCR (qPCR) was performed using the SYBR Green I‐based chimera fluorescence method on the Roche LightCycler 480 system to assess mRNA and miRNA expression levels. *β*‐Actin served as the internal control for mRNA, while U6 was used as the reference gene for miRNA quantification. Primers for the miR‐33 gene were designed using the tail‐adding method.

The primer sequences were as follows:

• *SREBP-1*: forward 5 ^′^‐CCCTGGTCTACCATAAGCTGC‐3 ^′^, reverse 5 ^′^‐CTTCACTCTCAATGCAGCCG‐3 ^′^
• *SREBP-2*: forward 5 ^′^‐CAACATTCAGCACCACTCCG‐3 ^′^, reverse 5 ^′^‐ACCAGGCTTTGGACTTGAGG‐3 ^′^
• *β-Actin*: forward 5 ^′^‐TGGCACCCAGCACAATGAA‐3 ^′^, reverse 5 ^′^‐CTAAGTCATAGTCCGCCTAGAAGCA‐3 ^′^
• *miR-33a*: forward 5 ^′^‐CAATGTTTCCACAGTGCATCAC‐3 ^′^
• *miR-33b*: forward 5 ^′^‐CAGTGCCTCGGCAGTGCA‐3 ^′^
• *U6*: forward 5 ^′^‐GGAACGATACAGAGAAGATTAGC‐3 ^′^, reverse 5 ^′^‐TGGAACGCTTCACGAATTTGCG‐3 ^′^

For SREBP mRNA detection, each 20 *μ*L reaction mixture contained 10 *μ*L 2X SYBR Green Pro Taq HS Premix, 2 *μ*L cDNA, 0.4 *μ*L each of forward and reverse primers, and 7.2 *μ*L RNase‐free water. For miR‐33 analysis, the 20 *μ*L reaction mixture consisted of 10 *μ*L 2X SYBR Green Pro Taq HS Premix II, 2 *μ*L cDNA, 0.8 *μ*L each of forward and reverse primers, and 6.4 *μ*L RNase‐free water.

The thermal cycling protocol included an initial activation at 95°C for 30 s, followed by 40 cycles of denaturation at 95°C for 5 s, annealing at 60°C for 30 s, and extension at 60°C for 30 s.

### 2.3. Criteria for Relevant Indicators

The criteria for diagnosing obesity among kids aged 3–6 years are derived from the WHO growth reference for weight‐for‐height in this age group. Obesity was defined as a weight exceeding the reference weight‐for‐height by more than 20%. The criteria for relevant indicators are as follows: Based on the diagnostic criteria from the National Center for Health Statistics/Centers for Disease Control and Prevention (NCHS/CDC), pathological and secondary obesity were excluded. Obesity was categorized as mild (20%–29%), moderate (30%–49%), and severe (≥ 50%) based on the percentage of body weight exceeding the height standard weight according to different ages to diagnose whether it is obesity.

According to the 2017 reference standards for blood pressure in Chinese children aged 3–17 years by gender, age, and height, a systolic or diastolic blood pressure ≥ P95 is classified as high blood pressure. In this study, it is necessary to calculate the P95 blood pressure cutoff point according to different ages to diagnose whether it is hypertension.

The evaluation of blood glucose in children follows the diagnostic criteria released by the American Diabetes Association (ADA) in 2020, where a fasting blood glucose level of ≥ 5.6 mmol/L is considered abnormal.

The evaluation of blood lipid levels in children refers to the “Expert Consensus on the Diagnosis and Treatment of Dyslipidemia in Children (2022)” issued by China. Dyslipidemia is defined as an abnormality in at least one of the following serum variables: TG, TC, LDL‐C, HDL‐C, or non‐HDL‐C. Specifically, dyslipidemia criteria include serum TG ≥ 1.12 mmol/L (0–9 years old) or ≥ 1.46 mmol/L (≥ 10 years old), serum TC ≥ 5.17 mmol/L, serum LDL‐C ≥ 3.36 mmol/L, serum HDL‐C < 1.03 mmol/L, or non‐HDL‐C ≥ 3.74 mmol/L.

## 3. Statistical Analysis

The statistical evaluation was conducted by employing SPSS 20.0. The findings from the nonnormally distributed metrics were articulated as median, and comparative analyses among the categories were conducted applying the Wilcoxon rank‐sum test. The strength of the relationship between the SREBP mRNA/miR‐33 expression levels variables and each of the biomarkers was quantified using nonparametric Spearman’s rank‐order correlation. SREBP mRNA was statistically analyzed with *β*‐actin as the internal reference gene, while miR‐33 expression level was statistically analyzed with U6 as the internal reference gene. Relative gene expression in qPCR was assessed employing the 2^−*Δ* *Δ*CT^ method: (1) The CT value of the reference gene served to normalize the CT value of the target gene through the calculation of relative expression levels: *Δ*CT (test) = CT (target, test) − CT (ref, test) *Δ*CT (calibrator) = CT (target, calibrator) − CT (ref, calibrator). (2) The *Δ*CT values from the calibration samples were employed to normalize the *Δ*CT values of the test samples: *Δ* *Δ*CT = *Δ*CT (test) − *Δ*CT (calibrator). (3) Expression level ratios were calculated as follows: Relative expression level *F* = 2^−*Δ* *Δ*CT^. A *p* value of < 0.05 indicated statistical significance.

## 4. Outcomes

### 4.1. Clinical and Biochemical Features

Table 1 shows the prenatal and parental characteristics of the two groups of children (ART and NC). There were no significant differences between the two groups in terms of gestational age, weight gain, body mass index, or mode of delivery. A total of 20 pregnant women in the ART group had complications, including 2 cases (2.04%) of placenta previa, 10 cases (10.20%) of premature rupture of membranes, 3 cases (3.06%) of placental adhesion, 1 case (1.02%) of eclampsia, and 4 cases (4.08%) of fetal distress. In the control group, a total of 18 pregnant women had complications, including 1 case (0.93%) of placenta previa, 5 cases (4.63%) of premature rupture of membranes, 4 cases (3.70%) of placental adhesion, 2 cases (1.85%) of eclampsia, and 6 cases (5.56%) of fetal distress. There is no clear difference between these two categories. The age of the parents of the children in the ART group was significantly higher than that of the children in the NC group. The analysis showed no significant differences between the two groups in terms of gender, birth height, weight, or postnatal hospitalization. The dietary structure of the children in the two groups was slightly different, and the daily dairy intake and vegetable intake frequency of children in the ART group were higher than those in the NC group. Table 2 and Figure 1 summarize the physical and blood pressure characteristics of the children in both groups. There were no significant differences in age, height, and systolic or diastolic blood pressure between the two groups. However, after adjusting for the age of the parents and the diet of the children, the weight of the children in the IVF group was significantly higher than that in the ICSI group and the NC group (*p* = 0.005 and *p* < 0.001, Figure 1a), the height of the children in the IVF group was significantly higher than that in the ICSI group (*p* = 0.007, Figure 1b), the BMI of the children in the IVF group was significantly higher than that in the NC group (*p* < 0.001, Figure 1c), and the waist circumference of the children in the IVF group was significantly higher than that in the ICSI group and the NC group (*p* = 0.021 and *p* < 0.001, Figure 1d).

**Table 1:** Perinatal features of the population of study.

**Characteristic**	**ART (** **n** = 98**)**	**NC (** **n** = 108**)**	**Z**/**χ** ^2^ **value**	**p** **value**
Gender (*n*, %)				
Male	59 (60.20)	60 (55.56)	0.455^b^	0.500
Female	39 (39.80)	48 (44.44)		
Maternal age at delivery (years)	33.00 (30.00, 35.00)	30.00 (27.00, 32.00)	−6.289^a^	< 0.001
Paternal reproductive age (years)	35.00 (32.00, 36.00)	30.00 (28.00, 34.00)	−6.708^a^	< 0.001
Paternal education level (*n*, %)				
Master’s degree or higher	28 (28.57)	35 (32.41)	−0.202^c^	0.840
College	62 (63.27)	61 (56.48)		
Senior high school or lower	8 (8.16)	12 (11.11)		
Maternal education level (*n*, %)				
Master’s degree or higher	19 (19.39)	28 (25.93)	−1.222^c^	0.222
College	68 (69.39)	71 (65.74)		
Senior high school or lower	11 (11.22)	9 (8.33)		
Maternal BMI (kg/m^2^)	23.15 (20.90, 24.88)	23.24 (20.67, 24.72)	−1.451^a^	0.147
Paternal BMI (kg/m^2^)	24.31 (21.36, 26.79)	23.09 (21.35, 25.95)	−0.564^a^	0.573
Gestational age at birth (weeks)	38.00 (37.20, 39.10)	38.10 (37.20, 39.20)	−1.076^a^	0.282
< 37 weeks (*n*, %)	18 (18.37)	18 (16.67)	0.103^b^	0.748
37–42 weeks (*n*, %)	80 (81.63)	90 (83.33)		
Weight gain during pregnancy (kg)	12.5 (10.50, 13.85)	12.5 (10.50, 13.98)	−0.443^a^	0.658
Pregnancy‐related comorbidities (*n*, %)				
Yes	20 (20.41)	18 (16.67)	0.478^b^	0.489
No	78 (79.59)	90 (83.33)		
Delivery method (*n*, %)				
Natural childbirth	55 (56.12)	73 (67.59)	2.873^b^	0.090
Cesarean section	43 (43.88)	35 (32.31)		
Birthweight (kg)	3.15 (2.80, 3.40)	3.20 (2.90, 3.50)	−0.488^a^	0.625
Birth length (cm)	50.0 (49.0, 50.63)	50.0 (49.5, 50.88)	−0.253^a^	0.800
Neonatal hospitalization treatment (*n*, %)				
Yes	11 (11.22)	8 (7.41)	0.894^b^	0.344
No	87 (88.78)	100 (86.01)		
Duration of breastfeeding, *n* (%)				
Never	2 (2.04)	3 (2.78)	−0.892^c^	0.372
0–3 months	6 (6.12)	10 (9.26)		
3–6 months	15 (15.31)	12 (11.11)		
7–12 months	30 (30.61)	23 (21.30)		
> 12 months	45 (45.92)	60 (55.55)		
Diet, exercise, and sleep conditions, *n* (%)				
Daily food dairy (300 mL or more)	54 (55.10)	75 (69.44)	4.515^b^	0.034
Fruit twice or more daily	77 (78.57)	85 (78.70)	0.001^b^	0.982
Vegetables twice or more daily	90 (91.84)	86 (79.63)	6.153^b^	0.013
Nuts once or more weekly	72 (73.47)	83 (76.85)	0.316^b^	0.574
Marine products once or more weekly	90 (91.84)	98 (90.74)	0.077^b^	0.781
Physical activity ≥ 2 h/day	91 (92.86)	96 (88.89)	0.966^b^	0.326
Sleep time ≥ 10 h/day	92 (93.88)	99 (91.67)	0.372^b^	0.542

*Note:* Continuous variables are denoted as median; *χ* ^2^ test or the Wilcoxon rank‐sum test was utilized to compare the variations between the two cohorts. When the expected count is less than 5, the Fisher exact test is used.

Abbreviations: ART, assisted reproductive technology; NC, naturally conceived.

^a^Wilcoxon rank‐sum test.

^b^ *χ* ^2^ test.

^c^Wilcoxon rank‐sum test.

**Table 2:** Physical development status of the study population.

**Characteristic**	**IVF (** **n** = 63**)**	**ICSI (** **n** = 35**)**	**NC (** **n** = 108**)**	**p** **(** **α** **) value**	**p** **(** **β** **) value**	**p** **(** **δ** **) value**	**Adjusted** **β**(**α**_1_) **(95% CI)**	**p** **(** **α**_1_ **) value**	**Adjusted** **β**(**β**_1_) **(95% CI)**	**p** **(** **β**_1_ **) value**	**Adjusted** **β**(**δ**_1_) **(95% CI)**	**p** **(** **δ**_1_ **) value**
Age (months)	59.00 (49.00, 65.00)	53.00 (47.00, 61.00)	57.50 (49.00, 66.00)	0.077	0.885	0.120	—	—	—	—	—	—
Weigh (kg)	20.00 (17.10, 23.40)	17.20 (16.10, 19.50)	17.40 (15.50, 20.10)	0.003	< 0.001	0.976	−0.29 (−2.80, −0.53)	0.005	−0.32 (−5.14, −1.60)	< 0.001	−0.06 (−2.31, 1.21)	0.540
Height (cm)	111.30 (105.10, 117.70)	106.10 (103.80, 112.20)	109.40 (104.13, 118.08)	0.008	0.125	0.105	−0.28 (−3.69, −0.59)	0.007	−0.15 (−4.83, 0.37)	0.093	−0.19 (−6.04, 0.07)	0.056
BMI (kg/cm^2^)	15.51 (14.72, 17.33)	15.19 (14.59, 16.14)	14.83 (14.09, 16.05)	0.122	< 0.001	0.138	−0.19 (−1.01, 0.03)	0.062	−0.31 (−2.34, −0.72)	< 0.001	0.14 (−0.21, 1.45)	0.141
WC (cm)	53.10 (49.50, 54.70)	50.20 (48.80, 53.80)	49.05 (47.70, 51.63)	0.042	< 0.001	0.015	−0.24 (−1.62, −0.14)	0.021	−0.41 (−4.17, −1.88)	< 0.001	−0.10 (−0.65, 2.10)	0.296
SBP (mmHg)	93.00 (90.00, 98.00)	96.00 (90.00, 98.00)	95.50 (90.00, 98.00)	0.467	0.327	0.987	0.00 (−1.72, 1.72)	1.000	0.04 (−2.13, 3.33)	0.667	−0.08 (−4.12, 1.77)	0.431
DBP (mmHg)	60.00 (58.00, 64.00)	60.00 (56.00, 62.00)	60.00 (58.00, 64.75)	0.342	0.595	0.089	−0.14 (−2.23, 0.46)	0.193	−0.07 (−3.01, 1.21)	0.400	−0.06 (−2.93, 1.52)	0.533

*Note:* Continuous variables are denoted as median. The Wilcoxon rank‐sum test was utilized to determine the variations among the cohorts. *α*, IVF group versus ICSI group; *β*, IVF group versus NC group; *δ*, ICSI group versus NC group. *α*_1_, IVF group versus ICSI group (adjusted for parents’ ages and children’s dietary conditions); *β*_1_, IVF group versus NC group (adjusted for parents’ ages and children’s dietary conditions); *δ*_1_, ICSI group versus NC group (adjusted for parents’ ages and children’s dietary conditions).

Figure 1Physical condition of children born with assisted reproductive technology (IVF group, *n* = 63; ICSI group, *n* = 35) and children born through natural conception (*n* = 108). (a) Comparison of the weight of children in the IVF group and the ICSI group (*p* = 0.005); comparison of the weight of children in the IVF group and the NC group (*p* < 0.001). (b) Comparison of height of children in the IVF and ICSI groups (*p* = 0.007). (c) Comparison of BMI between children in the IVF and NC groups (*p* < 0.001). (d) Comparison of waist circumference between children in the IVF and NC groups (*p* < 0.001). Comparison of waist circumference between the IVF and ICSI groups (*p* = 0.021).  ^∗^ *p* < 0.05;  ^∗∗^ *p* < 0.01;  ^∗∗∗^ *p* < 0.001.

**Figure:**
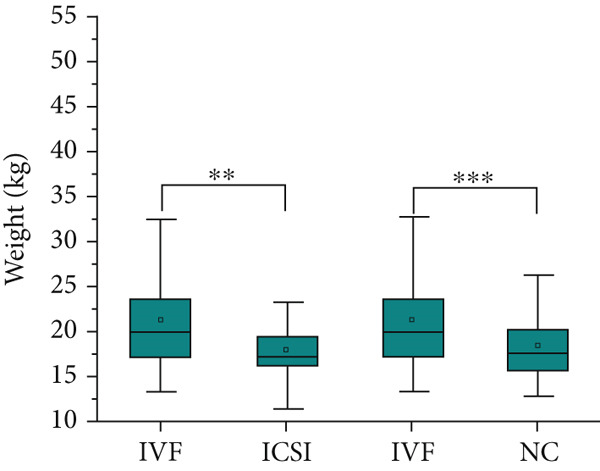
(a)

**Figure:**
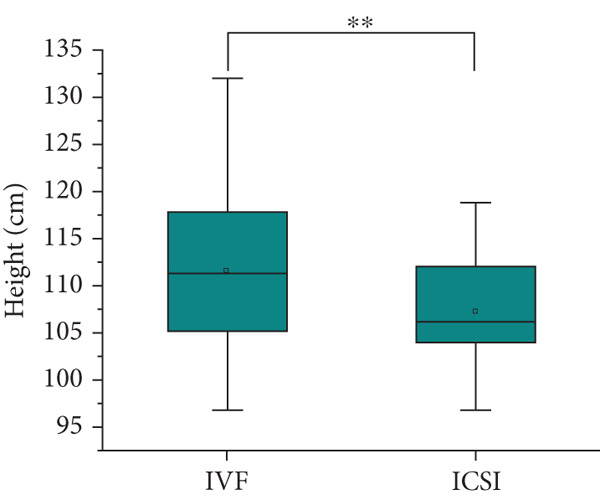
(b)

**Figure:**
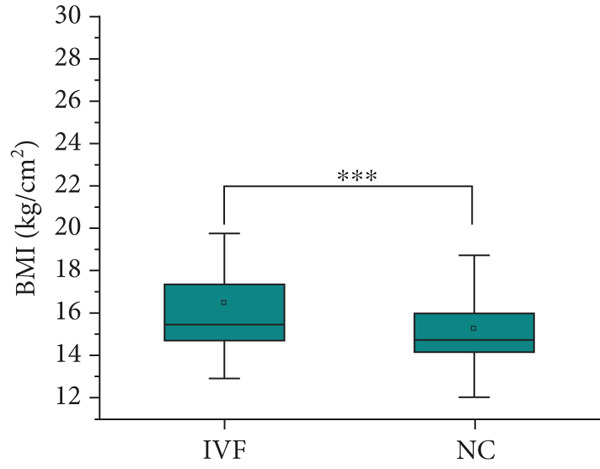
(c)

**Figure:**
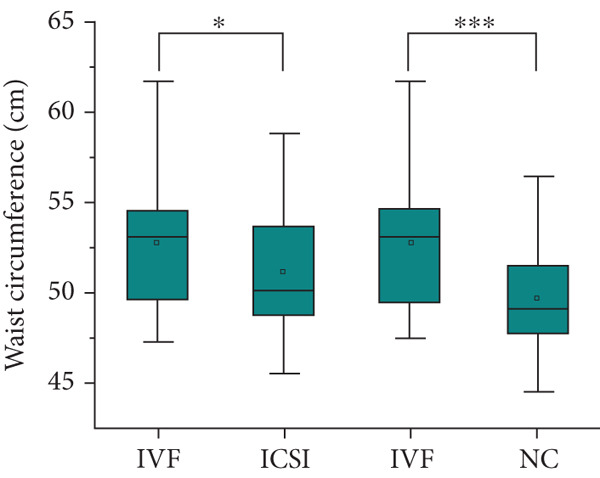
(d)

The analysis of blood lipids, blood glucose, and various biochemical parameters showed that the serum triglycerides in the ICSI group were significantly higher than those in the NC group (*p* = 0.015, Figure 2a), the HDH‐C in the IVF group was significantly lower than that in the NC group (*p* = 0.001, Figure 2b), and the ApoA1 in the IVF and ICSI groups was significantly lower than that in the NC group (*p* = 0.009 and *p* = 0.005, Figure 2c). The ApoB of children in the ICSI group was significantly higher than that in the IVF group and NC group (*p* = 0.039 and *p* = 0.006, Figure 2d), the fasting insulin in the ICSI group was significantly higher than that in the NC group (*p* = 0.006, Figure 2e), and the HOMA‐IR in the ICSI group was significantly higher than that in the IVF group and the NC group (*p* = 0.045 and *p* = 0.002, Figure 2f). There were no significant differences between the groups in other aspects (see Table 3 and Figure 2).

Figure 2Comparison of metabolic index status of children born through assisted reproductive technology (IVF group, *n* = 63; ICSI group, *n* = 35) and children born through natural conception (*n* = 108). (a) Serum triglycerides in the ICSI group and NC group were compared (*p* = 0.015). (b) Comparison of serum HDL cholesterol between children in the IVF and NC groups (*p* = 0.001). (c) Comparison of serum apolipoprotein A1 levels in children in the IVF and NC groups (*p* = 0.009). Serum apolipoprotein A1 levels in the ICSI and NC groups were compared (*p* = 0.005). (d) Serum apolipoprotein B levels in children in the IVF and ICSI groups were compared (*p* = 0.039). Serum apolipoprotein B levels in the ICSI and NC groups were compared (*p* = 0.006). (e) Comparison of fasting insulin in children in the ICSI and NC groups (*p* = 0.006). (f) Comparison of HOMA‐IR between the IVF and ICSI groups (*p* = 0.045). Comparison of HOMA‐IR between the ICSI group and NC group (*p* = 0.002).  ^∗^ *p* < 0.05;  ^∗∗^ *p* < 0.01;  ^∗∗∗^ *p* < 0.001.

**Figure:**
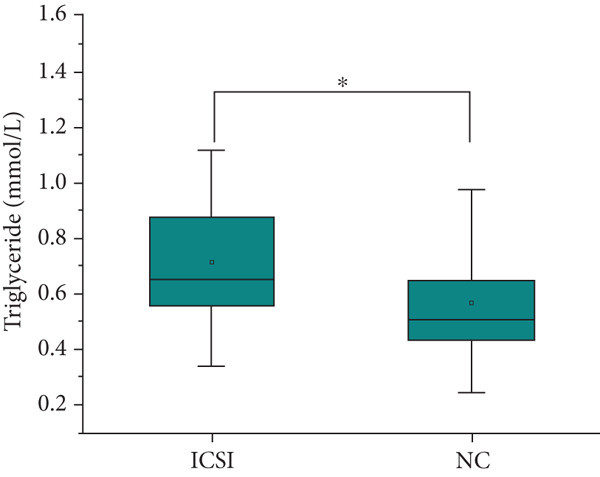
(a)

**Figure:**
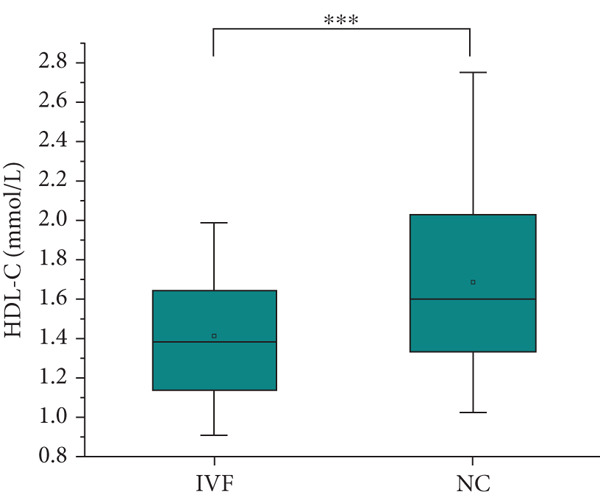
(b)

**Figure:**
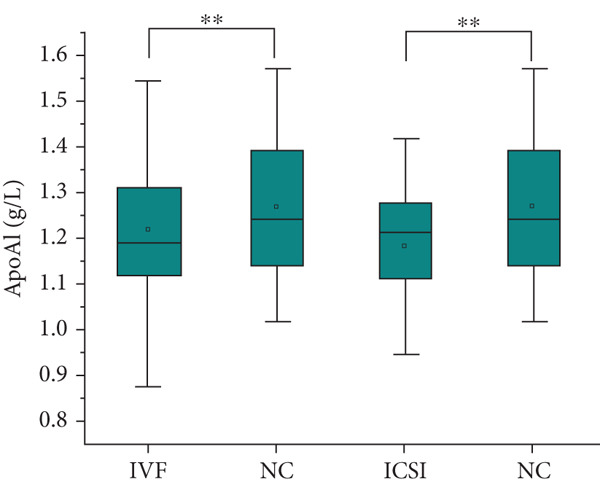
(c)

**Figure:**
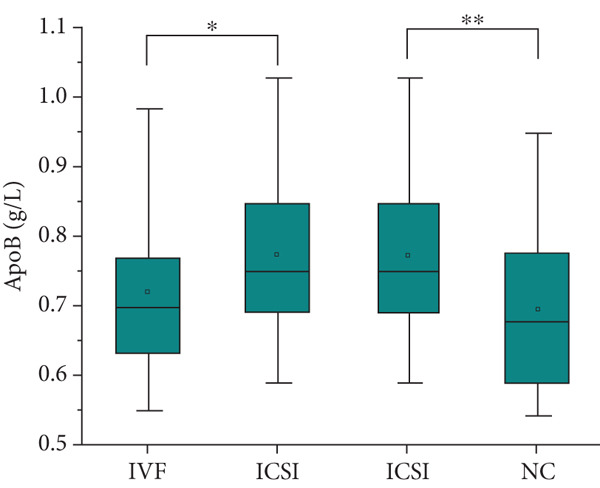
(d)

**Figure:**
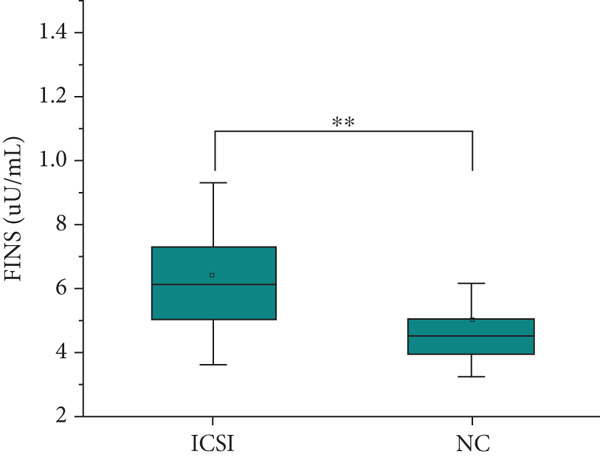
(e)

**Figure:**
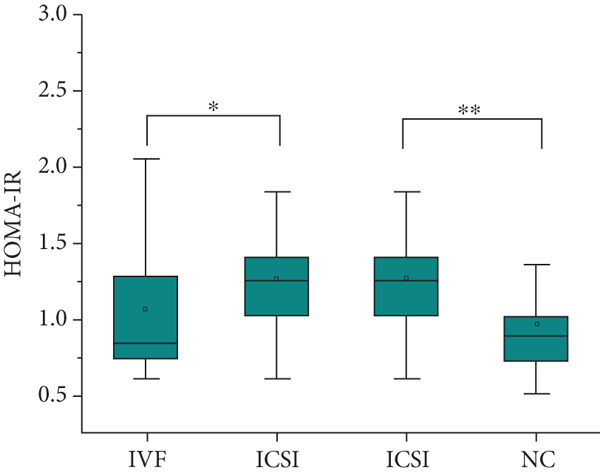
(f)

**Table 3:** Blood biochemical indicators status of the study population.

**Characteristic**	**IVF (** **n** = 63**)**	**ICSI (** **n** = 35**)**	**NC (** **n** = 108**)**	**p** **(** **α** **) value**	**p** **(** **β** **) value**	**p** **(** **δ** **) value**	**Adjusted** **β**(**α**_1_) **(95% CI)**	**p** **(** **α**_1_ **) value**	**Adjusted** **β** **(** **β**_1_ **) (95% CI)**	**p** **(** **β**_1_ **) value**	**Adjusted** **β** **(** **δ**_1_ **) (95% CI)**	**p** **(** **δ**_1_ **) value**
TC (mmol/L)	3.73 (3.34, 4.10)	4.04 (3.47, 4.25)	3.70 (3.26, 4.11)	0.090	0.687	0.031	0.10 (−0.07, 0.21)	0.318	−0.09 (−0.35, 0.10)	0.276	0.17 (−0.04, 0.54)	0.092
TG (mmol/L)	0.54 (0.43, 0.85)	0.65 (0.56, 0.87)	0.51 (0.43, 0.65)	0.068	0.160	< 0.001	0.14 (−0.02, 0.09)	0.156	−0.09 (−0.12, 0.04)	0.276	0.23 (0.02, 0.21)	0.015
HDL‐C (mmol/L)	1.38 (1.14, 1.64)	1.39 (1.30, 1.77)	1.61 (1.34, 2.02)	0.234	< 0.001	0.049	0.16 (−0.01, 0.11)	0.114	0.29 (0.11, 0.38)	0.001	−0.10 (−0.28, 0.08)	0.290
LDL‐C (mmol/L)	1.52 (1.28, 1.88)	1.53 (1.34, 1.74)	1.45 (1.26, 1.80)	0.714	0.254	0.192	0.00 (−0.12, 0.13)	0.977	−0.12 (−0.31, 0.05)	0.156	0.10 (−0.11, 0.32)	0.340
ApoA1 (g/L)	1.19 (1.12, 1.31)	1.21 (1.11, 1.28)	1.24 (1.14, 1.39)	0.516	0.060	0.025	−0.11 (−0.05, 0.01)	0.283	0.23 (0.02, 0.13)	0.009	−0.27 (−0.16, −0.03)	0.005
ApoB (g/L)	0.70 (0.63, 0.77)	0.75 (0.69, 0.85)	0.68 (0.59, 0.78)	0.010	0.203	< 0.001	0.22 (0.00, 0.05)	0.039	−0.14 (−0.07, 0.01)	0.108	0.26 (0.02, 0.12)	0.006
FBG (mmol/L)	4.23 (3.82, 4.70)	4.55 (3.97, 4.92)	4.37 (3.94, 4.67)	0.112	0.721	0.130	−0.15 (−0.03, 0.21)	0.149	−0.03 (−0.22, 0.16)	0.763	0.17 (−0.03, 0.42)	0.083
FINS (uU/mL)	4.76 (3.93, 5.60)	6.12 (5.03, 7.35)	4.58 (3.96, 5.06)	0.001	0.167	< 0.001	0.19 (−0.04, 0.88)	0.075	−0.12 (−1.23, 0.19)	0.152	0.26 (0.33, 1.95)	0.006
HOMA‐IR	0.85 (0.74, 1.27)	1.26 (1.03, 1.40)	0.89 (0.73, 1.01)	< 0.001	0.631	< 0.001	0.21 (0.00, 0.20)	0.045	−0.12 (−0.26, 0.04)	0.165	0.29 (0.10, 0.43)	0.002

*Note:* Continuous variables are denoted as median. The Wilcoxon rank‐sum test was utilized to determine the variations among the cohorts. *α*, IVF group versus ICSI group; *β*, IVF group versus NC group; *δ*, ICSI group versus NC group. *α*_1_, IVF group versus ICSI group (adjusted for parents’ ages and children’s dietary conditions); *β*_1_, IVF group versus NC group (adjusted for parents’ ages and children’s dietary conditions); *δ*_1_, ICSI group versus NC group (adjusted for parents’ ages and children’s dietary conditions).

### 4.2. Gene Expression Levels

The analysis showed no significant differences in abnormal blood pressure, blood glucose, and cholesterol levels between the two groups (*p* > 0.05). Nevertheless, changes in nutritional status were observed. The incidence of obesity and overweight was higher in the ART group than in the NC group. No abnormalities in fasting insulin and insulin resistance index were found in either group (Table 4).

**Table 4:** Evaluation of abnormal indicators across two groups of children.

**Parameter**	**ART (** **n** = 98**)**	**NC (** **n** = 108**)**	**Z**/**χ** ^2^ **value**	**p** **value**
Blood pressure status (*n*, %)				
Normal	97 (98.98)	108 (100.00)	1.107^a^	0.476
Abnormal	1 (1.02)	0 (0.00)		
Nutritional status (*n*,%)				
Obesity	15 (15.31)	4 (3.70)	−3.370^b^	0.001
Overweight	15 (15.31)	9 (8.33)		
Normal	68 (69.38)	95 (87.97)		
Blood lipid status (*n*, %)				
Normal	89 (90.82)	103 (95.37)	1.682^a^	0.195
Abnormal	9 (9.18)	5 (4.63)		
Blood sugar status (*n*, %)				
Normal	96 (97.96)	108 (100.00)	2.226^a^	0.225
Abnormal	2 (2.04)	0 (0.00)		

*Note:* The *χ* ^2^ test or the Wilcoxon rank‐sum test was utilized to determine the variations in the two cohorts. When the expected count is less than 5, the Fisher exact test is used.

^a^Wilcoxon rank‐sum test.

^b^ *χ* ^2^ test.

After adjusting for the age of parents and the diet of the children, the expression level of SREBP‐1 mRNA in the peripheral blood of children in the IVF group and ICSI group was significantly higher than that in the NC group (*p* = 0.003 and *p* < 0.001, Figure 3a), the expression level of SREBP‐2 mRNA in the ICSI group was higher than that in the NC group (*p* = 0.018, Figure 3b), and the expression level of miRNA‐33a in the peripheral blood of children in the ISCI group was lower than that in the IVF and NC groups (*p* = 0.008 and *p* = 0.045, Figure 3c), and miRNA‐33b in the IVF group was significantly higher than that in the NC group (*p* = 0.003, Figure 3d). There were no significant differences between the groups in other aspects, and the results are summarized in Table 5 and shown in Figure 3.

Figure 3Expression levels of SREBP mRNA and miRNA‐33 in the peripheral venous blood of children born with assisted reproductive technology (IVF group, *n* = 63; ICSI group, *n* = 35) and children born by natural conception (*n* = 108). (a) Comparison of SREBP‐1 mRNA expression levels in children in the IVF group and NC group (*p* = 0.003). The mRNA expression levels of SREBP‐1 in children in the ICSI and NC groups were compared (*p* < 0.001). (b) Comparison of SREBP‐2 mRNA expression levels between children in the ICSI group and NC group (*p* = 0.018). (c) Comparison of miRNA‐33a expression levels between children in the IVF and NC groups (*p* = 0.008). The expression levels of miRNA‐33a in children in the ICSI group and NC group were compared (*p* = 0.045). (d) Comparison of miRNA‐33b expression levels in the ICSI group and NC group (*p* = 0.003).  ^∗^ *p* < 0.05;  ^∗∗^ *p* < 0.01;  ^∗∗∗^ *p* < 0.001.

**Figure:**
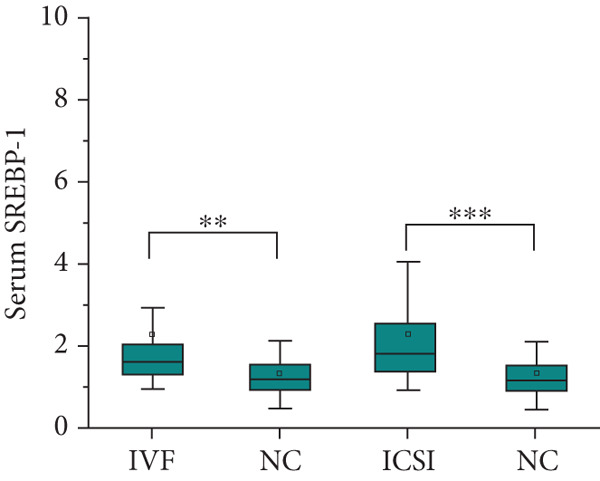
(a)

**Figure:**
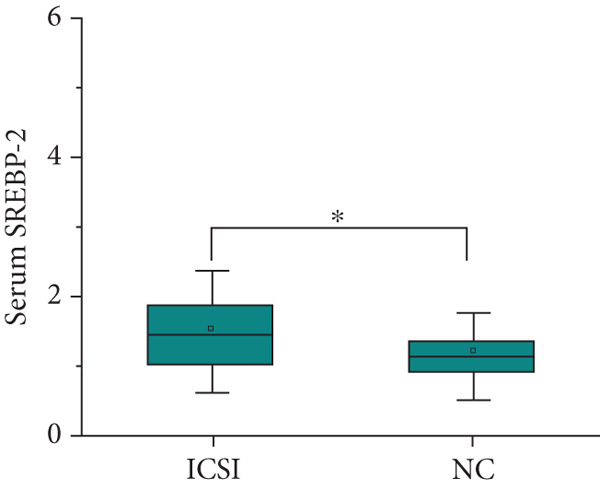
(b)

**Figure:**
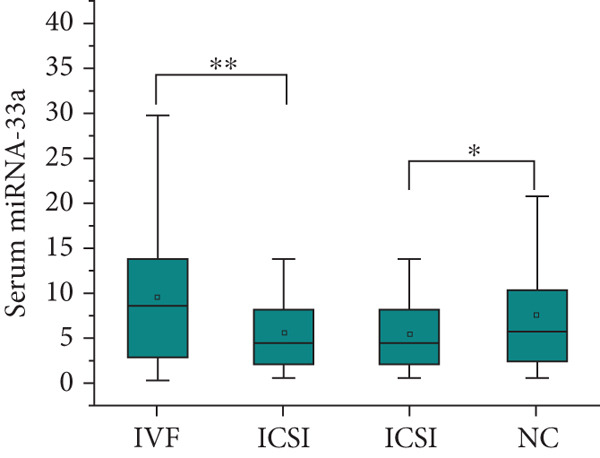
(c)

**Figure:**
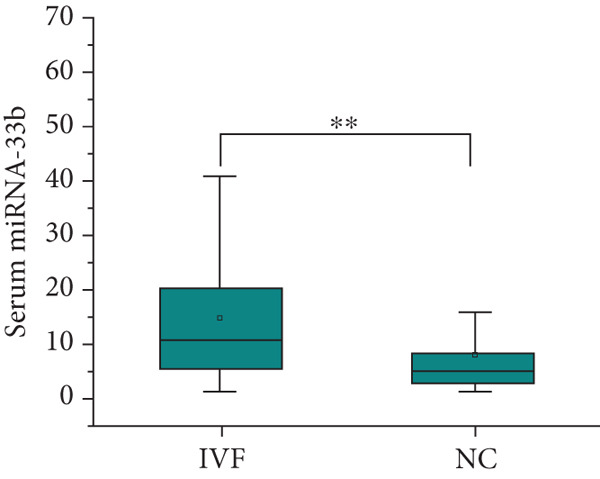
(d)

**Table 5:** Expression levels of SREBP mRNA and miRNA‐33 gene in peripheral venous blood of the study population.

**Characteristic**	**IVF (** **n** = 63**)**	**ICSI (** **n** = 35**)**	**NC (** **n** = 108**)**	**p** **(** **α** **) value**	**p** **(** **β** **) value**	**p** **(** **δ** **) value**	**Adjusted** **β** **(** **α**_1_ **) (95% CI)**	**p** **(** **α**_1_ **) value**	**Adjusted** **β** **(** **β**_1_ **) (95% CI)**	**p** **(** **β**_1_ **) value**	**Adjusted** **β** **(** **δ**_1_ **) (95% CI)**	**p** **(** **δ**_1_ **) value**
SREBP‐1 mRNA	1.61 (1.29, 2.00)	1.79 (1.38, 2.53)	1.14(0.92, 1.51)	0.174	< 0.001	< 0.001	0.00 (−0.54, 0.55)	0.980	−0.26 (−1.62, −0.34)	0.003	0.41 (0.59, 1.52)	< 0.001
SREBP‐2 mRNA	1.31 (1.05, 1.65)	1.42 (1.38, 2.53)	1.15 (0.94, 1.38)	0.186	0.018	0.003	0.19 (−0.01, 0.25)	0.063	−0.09 (−0.37, 0.10)	0.273	0.23 (0.07, 0.71)	0.018
miRNA‐33a	8.54 (3.01, 13.74)	4.43 (2.81, 8.34)	5.67 (2.34, 10.48)	0.022	0.095	0.235	−0.27 (−3.57, −0.55)	0.008	−0.14 (−4.58, 0.50)	0.115	−0.19 (−5.60, −0.06)	0.045
miRNA‐33b	9.99 (5.30, 19.86)	8.26 (5.68, 12.85)	4.88 (2.48, 8.18)	0.392	< 0.001	0.001	−0.14 (−4.26, 0.87)	0.193	−0.25 (−9.83, −2.08)	0.003	0.01 (−3.75, 4.12)	0.925

*Note:* Continuous variables are denoted as median. The Wilcoxon rank‐sum test was utilized to determine the variations among the cohorts. *α*, IVF group versus ICSI group; *β*, IVF group versus NC group; *δ*, ICSI group versus NC group. *α*_1_, IVF group versus ICSI group (adjusted for parents’ ages and children’s dietary conditions); *β*_1_, IVF group versus NC group (adjusted for parents’ ages and children’s dietary conditions); *δ*_1_, ICSI group versus NC group (adjusted for parents’ ages and children’s dietary conditions).

### 4.3. Relationship Between Clinical and Biochemical Factors and Gene Expression Levels

**Figure 4:**
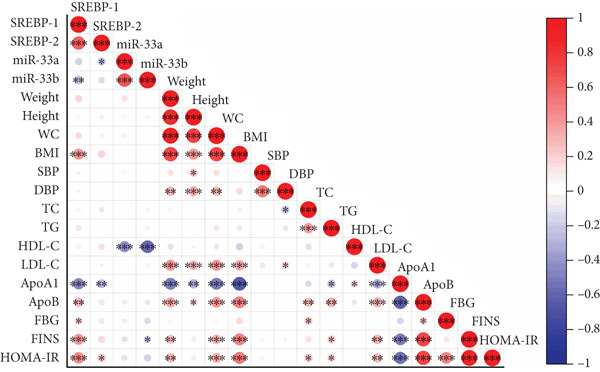
The correlation between SREBP mRNA and miRNA‐33 gene expression levels and physical and biochemical indicators. In this context, red stands for an affirmative link, while blue stands for a negative one. The intensity of the correlation is represented by the depth of the color: the darker the color, the stronger the correlation. Colorless areas signify that the correlation is not significant.  ^∗^ *p* < 0.05;  ^∗∗^ *p* < 0.01;  ^∗∗∗^ *p* < 0.001.

In the ART group, there was a positive correlation between the expression level of SREBP‐1 mRNA and BMI (*R* = 0.430, 95% CI [0.229–0.615]), ApoB (*R* = 0.316, 95% CI [0.114–0.504]), FBG (*R* = 0.210, 95% CI [0.002–0.424]), FINS (*R* = 0.435, 95% CI [0.256, 0.594]), and HOMA‐IR (*R* = 0.473, 95% CI [0.294, 0.639]) but negatively correlated with ApoA1 (*R* = −0.462, 95% CI [−0.635, −0.250]). The expression level of SREBP‐2 mRNA was positively correlated with HOMA‐IR (*R* = 0.251, 95% CI [0.057–0.434]) and negatively correlated with ApoA1 (*R* = −0.283, 95% CI [−0.465, −0.092]). The expression level of miRNA‐33a was inversely correlated with HDL‐C (*R* = −0.440, 95% CI [−0.600, −0.256]), while the expression level of miR‐33b was inversely correlated with HDL‐C (*R* = −0.580, 95% CI [−0.740, −0.382]) and FINS (*R* = −0.204, 95% CI [−0.405, −0.007]) (Table 6 and Figure 4).

**Table 6:** The correlation between the expression levels of SREBP mRNA and miRNA‐33 gene in peripheral venous blood and physical and biochemical indicators in the ART group (*n* = 98).

**Parameter**	**SREBP-1 mRNA**		**SREBP-2 mRNA**		**miRNA-33a**		**miRNA-33b**	
	**R**	**p**	**R**	**p**	**R**	**p**	**R**	**p**
Weight	0.187	0.065	0.014	0.895	0.145	0.153	−0.010	0.920
Height	0.070	0.493	−0.079	0.438	0.078	0.446	−0.068	0.503
BMI	0.430	< 0.001	0.189	0.063	0.057	0.574	−0.010	0.922
WC	0.165	0.104	−0.045	0.658	0.089	0.385	0.025	0.809
SBP	−0.044	0.664	−0.025	0.808	0.068	0.506	0.051	0.617
DBP	−0.052	0.614	0.004	0.967	0.014	0.890	0.057	0.575
TC	0.082	0.420	−0.105	0.302	0.009	0.930	0.080	0.432
TG	0.138	0.174	0.024	0.812	−0.003	0.973	−0.075	0.461
HDL‐C	0.099	0.332	0.155	0.127	−0.440	< 0.001	−0.580	< 0.001
LDL‐C	0.017	0.871	−0.057	0.578	0.031	0.759	0.088	0.389
ApoA1	−0.462	< 0.001	−0.283	0.005	−0.037	0.714	−0.028	0.781
ApoB	0.316	0.002	0.131	0.199	−0.094	0.356	−0.108	0.290
FBG	0.210	0.038	0.134	0.188	−0.081	0.430	−0.155	0.129
FINS	0.435	< 0.001	0.178	0.080	−0.129	0.206	−0.204	0.044
HOMA‐IR	0.473	< 0.001	0.251	0.013	−0.119	0.242	−0.191	0.059

*Note:* The Spearman (nonnormally distributed measurement data) analyzes correlations between variables.

Abbreviations: ApoA1, apolipoprotein A1; ApoB, apolipoprotein B; BMI, body mass index; DBP, diastolic blood pressure; FBG, fasting blood glucose; FINS, fasting insulin; HDL, high‐density lipoprotein; HOMA‐IR, homeostatic model assessment of insulin resistance; LDL, low‐density lipoprotein cholesterol; SBP, systolic blood pressure; TC, total cholesterol; TG, triglyceride; WC, waist circumference.

## 5. Discussion

Regarding perinatal status, many prior investigations indicate that children conceived via ART often experience less favorable perinatal outcomes relative to those developed via NC, including an elevated occurrence of premature birth and low birth weight [18–20]. By analyzing the perinatal‐related statistical data of the two groups of research subjects, we found that children in the ART group had significantly older parents compared to those in the NC cohort. No notable variations were detected in other aspects, including gestational age, maternal comorbidities during pregnancy, birth weight and height of the infants, and postnatal hospitalization status. These findings are inconsistent with most previous research results. The poor perinatal results observed in kids developed through ART, like preterm birth and low birth weight, could be partially attributed to the increasing occurrence of multiple births [2, 21–23]. Among the ART group population recruited in our current study, the vast majority were singleton pregnancies, with only three pairs of twin children and no pregnancies of three or more. This might be one of the reasons why the perinatal results of the two cohorts in the investigation showed no important variations.

By comparing the physical conditions, notable disparities were noted between the two cohorts of children regarding their weight, waist circumference, and BMI. Nevertheless, no notable disparities were observed in height and blood pressure between the two cohorts. Additionally, the rate of obesity was greater in the ART cohort relative to the NC cohort, consistent with previous findings [24–29]. The factors contributing to this may include the following reasons: (a) Parents of children conceived via ART generally demonstrate increased levels of attention and engagement in their children’s development [30], which may also extend to providing more pronounced support for their children’s dietary habits. (b) Obesity is a condition that typically does not present with immediate severe symptoms, which may influence the willingness to seek treatment. Parents of children conceived naturally may exhibit a higher propensity to seek treatment for their children’s obesity [25]. (c) Based on the DoHaD hypothesis—the origin of human health and disease development, an abnormal intrauterine environment may induce fetal “adaptation” mechanisms. This “adaptation” may result in IR and elevate the likelihood of obesity in the future [31, 32]. Finally, a variety of investigations have shown that epigenetic modifications are linked to ART [33, 34], and epigenetic mechanisms could be influential in the progression of obesity [35, 36]. A recent long‐term follow‐up study, from 3 months after the mother discovered her pregnancy until the child was 7 years old, regularly measured the height and weight of the children. Research indicates that children conceived via ART exhibited accelerated growth postnatally; however, at the age of 17, there existed no notable distinction between the two categories [37]. This indicates that children conceived via ART might demonstrate an increased rate of weight gain during the early stages of life, followed by a slower rate in later stages. As a result, there is a scenario in which early‐life factors like weight, waist circumference, BMI, and obesity incidence are greater in kids developed via ART than those developed via NC, but the variations between the two groups diminish or even disappear over time, requiring longer term follow‐up observation. Certainly, children conceived through ART warrant closer monitoring of indicators such as weight and waist circumference. Should any abnormalities be detected, prompt intervention is advisable to mitigate the potential increased risk of associated diseases.

Regarding the metabolism of glucose, the risks of diabetes and abnormal blood glucose regulation in offspring conceived through ART have remained a key focus of research; however, the conclusions remain inconsistent. On the one hand, some studies indicate that blood glucose measurement values in offspring conceived through ART are comparable to those of NC offspring, without an elevated likelihood of Type 2 diabetes [25, 38]. Conversely, certain research indicates that single‐embryo conception through frozen embryo transfer could be potentially attributed to a heightened likelihood of developing Type 1 diabetes [39, 40], that IVF‐ICSI offspring exhibit higher fasting insulin levels compared to NC offspring [41], and that HOMA‐IR values are elevated in children conceived through ART [42, 43]. In addition, studies have demonstrated that there are differences in glucose metabolism regulation between infants created by ART and the ones conceived by NC [44]. Offspring conceived with ART show a higher incidence of resistance to insulin and Type 2 diabetes in adulthood [28]. A comparison was conducted of FBG, FINS, and HOMA‐IR across the two groups of preschool children and observed that there was no empirically important distinction in FBG amounts. Nonetheless, the children within the ART group demonstrated elevated levels of FINS and HOMA‐IR values. This aligns with the results of a case‐control study that examined 380 children conceived via ART alongside those conceived via natural means [8]. While variations in FINS and HOMA‐IR were noted between the two categories of children, the assessed values of FBG, FINS, and HOMA‐IR in both cohorts predominantly fell within the typical range. No abnormal glucose metabolism or incidence of diabetes was detected in either the recruited children in the ART group or those conceived naturally. Therefore, glucose metabolism in children conceived through ART appears to be normal before school age; however, closer monitoring of glucose metabolism‐related indicators may be warranted compared to naturally conceived children.

Regarding lipid metabolism, a comparison revealed no significant variations in TC and LDL‐C amounts among the two cohorts of children. However, notable variations were detected in TG, HDL‐C, ApoA1, and ApoB. Specifically, the ART group showed heightened amounts of TG and ApoB, while demonstrating reduced amounts of HDL‐C and ApoA1. Cui et al. [8] conducted a comparative analysis between kids developed via ART and the ones developed via NC, revealing that serum TC levels were markedly elevated in the former group, whereas ApoA amounts were notably diminished. Additionally, a multitude of studies have illustrated the variations in TC, TG, HDL‐C, LDL‐C, and ApoB levels among children conceived through ART and those conceived through natural means [38, 45–48]. These research results have similarities with ours. Furthermore, after statistical analysis, nine instances of dyslipidemia were observed within the ART cohort, while five instances were noted in the NC cohort. There was not a statistically significant distinction between the two sets, suggesting negligible variations in the occurrence of dyslipidemia in the two cohorts. Therefore, the occurrence of abnormal lipid metabolism in children conceived through ART fails to show a significant increase before school age.

In order to explore the underlying factors contributing to the discrepancies in specific metabolic indicators between the ART and the NC groups, we conducted an analysis of the expression levels of pivotal genes that monitor glycolipid metabolism. This included examining SREBP mRNA and miR‐33, which is derived from the intron splicing of SREBP mRNA, in the blood samples from both groups [11, 14, 16, 49–51]. SREBPs serve an essential function in regulating fatty acid synthesis and serve as the core regulatory factors for lipid homeostasis [52, 53]. The SREBP family comprises three subtypes: SREBP‐1a, SREBP‐1c, and SREBP‐2. The SREBP proteins are produced by the genes SREBP‐1 and SREBP‐2. In particular, SREBP‐1c and SREBP‐1a are products of the SREBP‐1 gene and serve an essential function in modulating the expression of genes that govern the production of fatty acids and TGs. Conversely, SREBP‐2 is synthesized from the SREBP‐2 gene and primarily governs the expression of genes linked to the production of cholesterol, including 3‐hydroxy‐3‐methyl glutaryl coenzyme A reductase (HMGCR) [53–57]. We conducted an analysis of the expression levels of SREBP‐1 mRNA and SREBP‐2 mRNA in the peripheral blood of children belonging to both the ART and the NC groups. The findings indicated that the expression levels of SREBP‐1 mRNA and SREBP‐2 mRNA in the peripheral blood of kids developed via ART were markedly elevated relative to those in kids developed via NC. In the ART cohort, the mRNA expression level of SREBP‐1 exhibited a positive correlation with BMI, ApoB, fasting blood glucose, fasting insulin, and the insulin resistance index, whereas it demonstrated a negative correlation with ApoA1. Furthermore, the expression level of SREBP‐2 mRNA exhibited a negative correlation with ApoA1. The findings indicate that the differential expression levels of SREBP mRNA may represent an important factor contributing to the differences in certain lipid indicators between the two groups of children and might potentially explain the greater occurrence of obesity observed in the ART cohort. Currently, there appears to be a scarcity of investigations examining the expression levels of SREBP mRNA in the peripheral blood of kids conceived through ART. Nevertheless, analogous conclusions have been drawn from relevant animal experiments. Laren et al. conducted a long‐term study on mice conceived through ART (from the embryonic stage to adulthood) and observed that SREBP‐2 exhibited higher expression levels (both transcripts and associated proteins) in female mice conceived via IVF. In 12‐week‐old male mice, the SREBP‐1 mRNA level in the ART cohort was considerably higher than in the NC cohort. Additionally, in 12‐week‐old IVF female mice, both SREBP‐2 mRNA and HMGCR were upregulated, indicating an early disruption of the cholesterol biosynthesis pathway, which persisted into adulthood [29]. In the study by Fang et al. on mice conceived through ART, female mice in the ART group showed substantially lower levels of Apo‐A1 and HDL‐C, along with a significant increase in LDL‐C levels. Further investigations revealed that, compared to the NC group, INSIG1 mRNA, SCAP mRNA, SREBP‐1 mRNA, and SREBP‐2 mRNA were significantly upregulated in ICSI mice at both 3 weeks and 10 weeks postbirth (2–12 times, *p* < 0.01). Additionally, at 1.5 years of age, INSIG1 mRNA, SCAP mRNA, SREBP‐1 mRNA, and SREBP‐2 mRNA were also significantly upregulated in both ICSI and IVM/ICSI mice (2–4 times, *p* < 0.01) [9]. Upregulation of SREBP‐1 mRNA expression enhances the expression of genes encoding FASN and ACC, which improves fatty acid production. When ACC is overexpressed, it regulates fat formation since it is the rate‐limiting enzyme for fatty acid production. FASN, an enzyme of multifaceted functionality, facilitates the de novo biosynthesis of long‐chain saturated fatty acids from acetyl‐CoA and malonyl‐CoA, playing a crucial role in the production of fatty acids [58]. Additionally, the upregulation of SREBP‐1 mRNA can lead to the overexpression of SREBP‐1c that fosters neofat synthesis and lipid accumulation in the liver. This subsequently induces insulin resistance, ultimately resulting in elevated FBG levels and dyslipidemia [59, 60]. The upregulation of SREBP‐2 mRNA expression increases HMGCR synthesis. HMGCR functions as the pivotal enzyme in the manufacturing of cholesterol, affecting both the rate of cholesterol production and the overall cholesterol concentrations within the organism. Increased levels of HMGCR in the liver may lead to a rise in systemic cholesterol levels within the bloodstream [57]. Studies have demonstrated that inhibiting SREBP‐1 mRNA expression reduces cholesterol levels, alleviates hepatic steatosis, and improves adipose tissue hypertrophy, obesity, and insulin resistance. Furthermore, suppressing SREBP‐2 mRNA expression decreases triglyceride levels [52, 61].

The generation of fat is regulated by a variety of adipocyte‐selective miRNA and transcription factors, which play roles in modulating blood lipid levels, macrophage differentiation, insulin resistance, and other processes. Consequently, miRNAs, as epigenetic regulators, can serve as tools for identifying new gene transcription pathways and potential therapeutic targets [62]. The miRNA‐33 gene is generated through the intron cleavage of the SREBP gene. People possess two miRNA‐33 genes: miRNA‐33b, found in the 17th intron of the SREBP‐1 gene on Chromosome 17, and miRNA‐33a, situated in the 16th intron of the SREBP‐2 gene on Chromosome 22. The mature forms of miRNA‐33a and miRNA‐33b exhibit a difference of merely two nucleotides while targeting the same entity [63]. MiR‐33 serves as a crucial regulatory element in the processes of lipid metabolism [64]. It regulates the reverse cholesterol transport process to control cholesterol levels and also plays a role in modulating insulin signaling pathways [65–70]. This time, we selected peripheral blood miR‐33 as another key gene involved in glycolipid metabolism regulation for determination and analysis, aiming to elucidate its role in glycolipid metabolism in children conceived through assisted reproduction. Through the analysis of miR‐33 expression levels in peripheral blood across the two groups, we observed little variation in the expression level of miR‐33a among the children in both groups. Nonetheless, there was a notable rise in the expression level of the miR‐33b gene in the peripheral blood of children within the ART group. Additionally, the expression level of miR‐33a exhibited an inverse relationship with HDL‐C, whereas the expression level of miR‐33b demonstrated an inverse relationship with both HDL‐C and FINS. At present, there is a scarcity of studies focused on the identification of the miR‐33 gene in children conceived via ART. However, multiple investigations have demonstrated that miR‐33a/b is capable of targeting genes responsible for fatty acid oxidation and HDL production, therefore altering systemic lipid homeostasis [71, 72]. MiR‐33 primarily regulates blood lipid levels through the modulation of ABCA1 gene expression [73, 74]. The upregulation of miR‐33b in the body restricts ABCA1 transcription, leading to reduced cholesterol efflux and consequently decreasing the formation of HDL‐C and ApoA1 [75]. Investigations have shown that short‐term therapy with miR‐33 inhibitors can considerably raise plasma HDL‐C concentrations. Consequently, it is hypothesized that the serum HDL‐C amounts in kids from the ART cohort were diminished compared to kids in the NC cohort, with the miR‐33 gene (notably miR‐33b) potentially playing a significant role in modulating this phenomenon. From the findings discussed, it makes sense to conclude that the increased expression of SREBP mRNA and miR‐33 may serve an essential function in the variations observed in glycolipid metabolism indicators between children in the ART and the NC groups. Nonetheless, the underlying mechanisms require further investigation. For this demographic, it is imperative to focus more intently on their metabolic wellness, especially in the pivotal phases of growth, development, and familial nurturing. A balanced diet and increased physical activity should be emphasized, while targeted child healthcare interventions are implemented. Regular monitoring of metabolic indicators, such as weight, blood lipids, and blood glucose, is also essential. In addition, current studies have shown that blood lipid levels progressively increase with age until adolescence and subsequently decline gradually [32]. Nonetheless, it is still uncertain if variations in the patterns of blood lipid alterations will manifest between the two groups of children moving forward. Consequently, it is essential to conduct continuous monitoring for these children, particularly those in the ART group, with dynamic monitoring of their metabolic changes extending into adolescence and potentially adulthood. In the end, causal and mechanistic terminology remains prevalent, despite lacking validation at the protein level or longitudinal studies. Nonetheless, this study has some limitations. First, this study is a cross‐sectional case‐control study, and the number of participants in this investigation is notably limited, especially within the experimental group, potentially impacting the broader applicability of the results. Expanding the research sample size is crucial in future studies to draw more accurate and reliable conclusions. Second, the follow‐up period for the study population was relatively short. Variations in both groups of children were noted solely prior to reaching school age, while the dynamic changes in related indicators remain unclear. Therefore, long‐term follow‐up of this study population until adolescence and even adulthood is necessary to better understand the long‐term trends in metabolic indicators and the expression levels of relevant genes. Third, due to the limitations of the data, this study did not perform a stratified analysis of gender, and of course, gender may have an impact on the outcome, which is a limitation of this study. Finally, although we determined the expression levels of key genes (SREBP mRNA and miR‐33) and elucidated their roles in metabolism, the investigation of underlying mechanisms (e.g., related proteins and enzymes) was not sufficiently in‐depth, warranting further exploration.

In conclusion, the current findings suggest that the health status of preschool‐age children conceived via modern ART is satisfactory, with an insignificant rise in the incidence of abnormal glucose and lipid‐related diseases noted. Nevertheless, in specific areas of metabolism, such as the prevalence of obesity, there was a greater occurrence among children in the ART cohort. Additionally, differences were observed in metabolic indicators such as triglycerides, HDL‐C, ApoA1, ApoB, FINS, and the HOMA‐IR of the ART and NC groups. These differences may be attributed to the elevated expression of SREBP‐1 mRNA, SREBP‐2 mRNA, and miR‐33b, although the underlying mechanisms require further investigation. The long‐term health outcomes of kids developed via ART hold considerable significance for society, families, and individuals alike.

## Ethics Statement

The Ethics Committee of Dalian Women and Children’s Medical Group issued approval for this investigation (Approval No. 2023007). Informed consent in writing was secured from the guardians of all participants who were minors.

## Consent

The authors declare that they have obtained written consent from the patients/guardians.

## Conflicts of Interest

The authors declare no conflicts of interest.

## Author Contributions

Contribution description: Y.C., D.H., and X.X. X.W. assisted in the study layout. Y.C. carried out an analysis of the data. Each author played a role in the recruitment of participants and the collection of blood samples. Y.C. drafted the initial manuscript. Y.C., P.Z., and X.W. played a significant role in the comprehensive revision of the article. All contributors examined and endorsed the final iteration of the manuscript. Y.C. and Y.W. have equal contributions.

## Funding

This study received support from the doctoral initiative at Dalian Women and Children’s Medical Group.

## Supporting information


**Supporting Information** Additional supporting information can be found online in the Supporting Information section. Supporting information 1 Raw data.

## Data Availability

All data generated or analyzed during this study are included in this published article and its supporting information files.
